# Supplementary material for: Influence of Drought Stress on the Rhizosphere Bacterial Community Structure of Cassava (Manihot esculenta Crantz)
Source: Int J Mol Sci. 2024 Jul 3;25(13):7326. doi: 10.3390/ijms25137326 (PMC11242396; doi:10.3390/ijms25137326)
Supplement: Supplementary file 1 [file ijms-25-07326-s001.zip › Supplementary Figure S1.pdf]

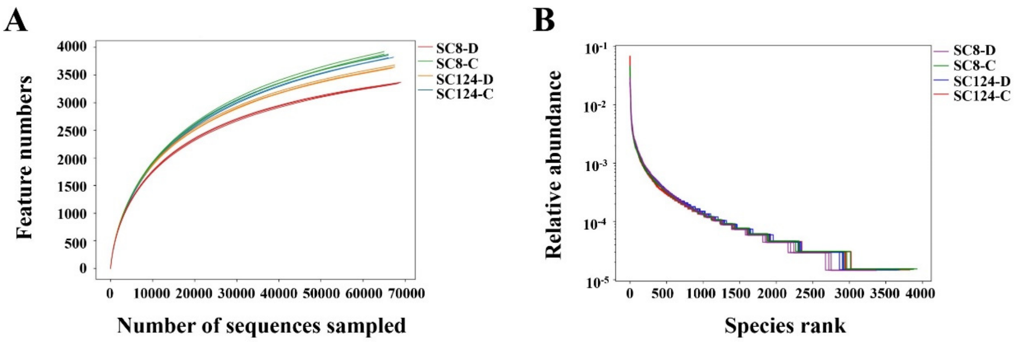

Supplementary Figure S1 Rarefaction curve and rank abundance curve of cassava rhizosphere soil samples in drought resistant group and water sensitive group under drought treatment
