# Supplementary material for: Influence of Drought Stress on the Rhizosphere Bacterial Community Structure of Cassava (Manihot esculenta Crantz)
Source: Int J Mol Sci. 2024 Jul 3;25(13):7326. doi: 10.3390/ijms25137326 (PMC11242396; doi:10.3390/ijms25137326)
Supplement: Supplementary file 1 [file ijms-25-07326-s001.zip › Supplementary Figure S2.pdf]

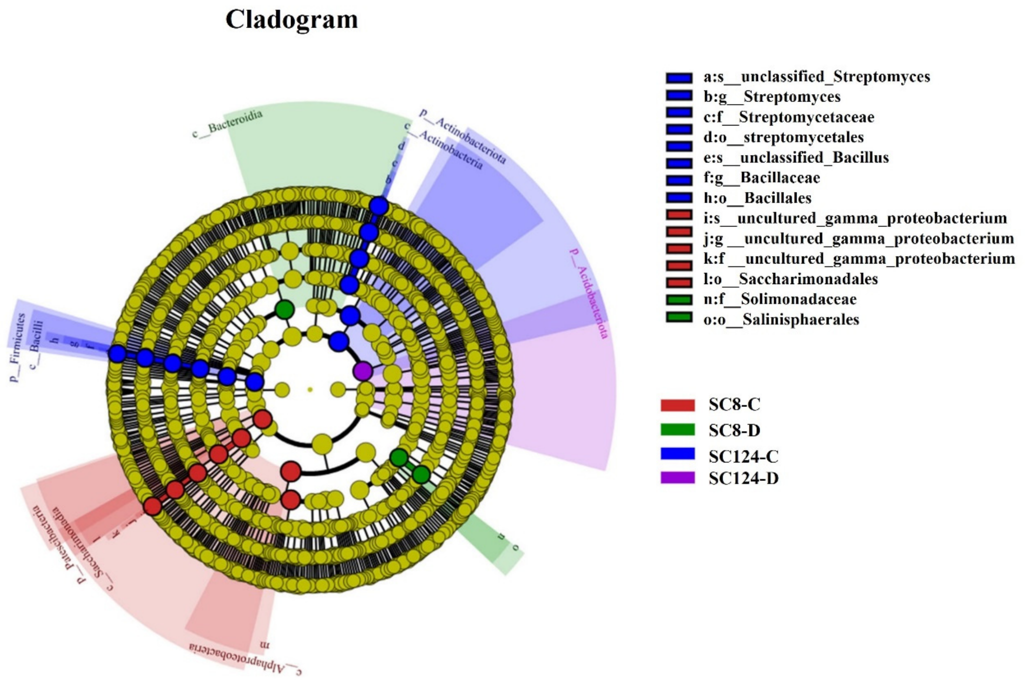

Supplementary Figure S2 LEfSe analysis of rhizosphere soil bacteria in different cassava varieties under drought stress (LDA score:4.0). Circles indicated phylogenetic levels from phylum to genus, the diameter of each circle was proportional to the abundance of the group; Different prefixes indicated different levels different levels (p: Phylum; c: Class, o: Order; f: Family; g: Genus. The same as below).
